# The Synergy of Legal and Medical Palliative Care: Challenges and Opportunities in Palliative MLP and the Yale Experience

**DOI:** 10.1017/jme.2023.161

**Published:** 2023

**Authors:** Rebecca Iannantuoni, Emily B. Rock, Abbe R. Gluck

**Affiliations:** 1: DAY PITNEY LLP, NEW HAVEN, CT, USA; 2: YALE LAW SCHOOL, NEW HAVEN, CT, USA

**Keywords:** Palliative Care, Medical-Legal Partnership

## Abstract

Palliative care and medical-legal partnership are complementary disciplines dedicated to integrating care to treat the whole patient and intervening before a legal or medical issue is at a crisis point. In this paper, we discuss the founding and operations of the Yale Palliative Medical Legal Partnership, give examples of typical cases, explain special considerations in this area of law, and propose areas for further research.

## I. Palliative Care and Medical-Legal Partnership

The missions of palliative care clinicians and of lawyers who focus on end of life planning have long been complementary. The medical-legal partnership (“MLP”) model provides a significant opportunity to advance these synergies. Palliative care is a broad approach encompassing the holistic treatment of patients with life-limiting illness, not just those at the end of life. A palliative care team works to “improv[e] pain and other symptom control, clarify[] goals of care, and guid[e] treatment decisions to meet those goals.”^1^ It is “an integrative perspective, which aims to transcend the sequestration of care that specialization created.”^2^ Palliative care is organically interdisciplinary: “[t]he myriad issues faced by a patient with a life-threatening illness, and a family who must adapt to the loss of one of its members, exceeds the expertise of any one care-giver.”^3^ Social workers have long been part of palliative care teams, along with doctors, nurses, clergy, and other providers. Palliative Medical Legal Partnership, or “PMLP,” adds lawyers to the team, recognizing the stressors of legal needs at the end of life — from guardianship to wills, powers of attorney, and other advance planning documents — and how addressing them furthers the mission of palliative care. In this article, we discuss the founding and operations of the Yale PMLP, give examples of typical cases, explain special considerations in this area of law, and propose areas for further research.

### A. What is a PMLP?

Legal issues abound in palliative care. Patients confronting their life-limiting illness may ask their medical providers questions that the provider is not trained to answer (e.g., what will happen to their minor children after their death? Do they need a will or power of attorney? Are they eligible for disability benefits?).^4^ Providers often feel frustrated that they cannot help when such questions are raised, and that they do not have a way to connect patients to legal services. The concept of a palliative-care medical-legal partnership embraces the same core philosophy as palliative care itself: making a patient comfortable and reducing their distress can improve health, extend lifespan, and enable a better end-of-life experience. PMLP reduces potential barriers to care by addressing a patient’s legal distress as part of their palliative care treatment plan. Patients gain peace of mind and comfort through working with a dedicated, competent legal team to help put their affairs in order.

Social workers have long been part of palliative care teams, along with doctors, nurses, clergy, and other providers. Palliative Medical Legal Partnership, or “PMLP,” adds lawyers to the team, recognizing the stressors of legal needs at the end of life — from guardianship to wills, powers of attorney, and other advance planning documents—and how addressing them furthers the mission of palliative care. In this article, we discuss the founding and operations of the Yale PMLP, give examples of typical cases, explain special considerations in this area of law, and propose areas for further research.

Unmet legal needs can affect patients’ health and the progression of their illness.^5^ This is especially true of the palliative care population, for whom “delays in resolution of exigent legal matters can result in poor coping, exacerbate social/legal issues, and ultimately add to illness-related suffering.”^6^ Palliative care and medical-legal partnerships share core values that make them naturally synergistic. “Both fields are rooted in patient and family advocacy, with access to quality, comprehensive care for vulnerable patients as an overarching priority, and both fields have emerged from innovations that challenge traditional paradigms of healthcare delivery.”^7^

In contrast to the “care fragmentation” that characterizes much of healthcare, MLP and palliative care are both dedicated to integrating care across disciplines, with an eye to treating the whole patient, not just one issue or symptom.^8^ MLP and palliative care also share the goal of intervening before a legal or medical issue is at a crisis point. Early consultation with PMLP “can assist patients upstream of crisis to help anticipate and proactively resolve health-related legal issues,” helping to replace deathbed, crisis-driven decision-making with thoughtful and proactive planning.^9^

### B. The Yale PMLP

The Yale PMLP is one of the few MLPs in the palliative care space. It engages the efforts of a palliative care social worker at Yale New Haven Hospital (YNHH), a lawyer from the community donating pro bono hours, and lawyers and law students from the Yale Law School (YLS) Medical-Legal Partnership program. Social workers play a crucial role in conducting a comprehensive social assessment of each new patient in need of assistance. Yale’s Gena Lennon-Gomez, MSW, LCSW, says “[w]ithin that assessment, we’re understanding the family composition, their needs, their financial picture, and what’s worrying them — what’s causing that existential distress.”

After the assessment, the social workers refer appropriate patients to PMLP. The legal supervisor reviews each referral to determine initial eligibility and then assigns a law student to initiate contact with the patient and collect the information necessary to assess the legal needs. These student-patient discussions yield insight into the patient’s priority of concerns.^10^ The student will then present, at a meeting of all PMLP students and supervisors, the issues and needs of the patient. The team triages and collectively develops a proposed plan of action. Then, with a supervising attorney’s oversight, the student will discuss the plan with the patient, draft the necessary documents, and take whatever other steps are required to meet the patient’s legal needs.

PMLP now receives about thirty referrals each year. Since the inception of the program, the Yale PMLP has provided legal assistance to over 200 palliative care patients. The most common legal issues are estate planning (e.g., wills or trusts), guardianship for minor children or other dependents, and educating and assisting in applications for public benefits programs. Some patients have a discrete issue to address, while others have legal needs requiring sustained engagement over the course of weeks or months. Students and supervisors spend significant time with each patient, often getting to know them and their families on a deeply personal level.

## II. Opportunities and Challenges in Starting A PMLP

The Yale PMLP was born in 2013-2014 out of a collaboration between Yale Law School Professor Abbe R. Gluck, who directs the Solomon Center for Health Law and Policy at Yale, and Yale New Haven Hospital’s Dr. Andrew Putnam, Director of Palliative Pain and Symptom Management and Assistant Professor of Clinical Medicine. Putnam, a palliative care specialist, had become increasingly frustrated and concerned by his team’s inability to help their patients address stressful legal needs. Care teams often find themselves making fraught and frantic calls to lawyers or to the hospital’s General Counsel’s office, for whom direct legal services are outside the scope of their job description and expertise. The PMLP helped fill in this gap.

An initial challenge to starting an MLP is overcoming potential trust issues between legal and medical professionals, who can be more accustomed to being adversaries than collaborators, as in medical malpractice cases.^11^ These “trust” challenges are heightened in the context of palliative care, which requires extreme sensitivity in dealing with patients and their families making decisions about end-of-life care, and where hospitals already have formal procedures to address these issues. A hospital’s general counsel’s office and/or ethics board may initially raise concerns that PMLP could interfere with the hospital’s established process for end-of-life decision-making. Making clear at the outset that PMLP does not engage with the medical team’s work or decisions about whether or how to prolong life is critically important to building trust.

At Yale, when PMLP was founded, two other MLPs had already been established in conjunction with Yale Law School, so trust relationships, while new, were already being developed. Nevertheless, a memorandum of understanding between the MLP and the hospital, with defined procedures about referrals and access to patients, helped smooth the way to launch the PMLP. smooth the way. The medical and legal partners to the PMLP should also clearly delineate that the patient’s relationship with their PMLP legal team is separate and distinct from their relationship with their medical providers. Patients need to understand that the PMLP works with the hospital but is not employed *by* the hospital.

Developing a meaningful referral process by which patients are referred to PMLP is also a significant component to minimizing skepticism. As at Yale, the general counsel’s office may initially want to review all referrals; over time, as trust builds, PMLP will likely manage referrals on its own. At Yale, the benefits derived from PMLP continue to spur the development of additional MLPs. The Geriatric and Oncology MLPs are recent additions to Yale Law’s MLP program that arose from the success of PMLP.

## III. Case Studies and Common Issues

The following case studies offer examples of the kind of work in which PMLP engages and its impact on patients as part of their palliative care treatment plan:

### A. Ms. A

Ms. A was initially referred to PMLP shortly after her brain cancer diagnosis. The PMLP team met with her at her home. They discussed her attempts to modify her mortgage, her need for a financial power of attorney and the potential guardianship issues for her then 15-year-old son. Notwithstanding several attempts to follow up with Ms. A after this meeting, she was silent. This is not an uncommon occurrence in the PMLP process: patients recognize they have legal needs but are unable to actually follow through on resolving them for a variety of reasons (e.g., they are too overwhelmed with treatment protocols, or they have not reached a point of acceptance).

About a year after the initial contact, the PMLP supervisor was contacted by Ms. A’s sister. Ms. A’s prognosis had changed and as a result, Ms. A was ready to revive the process. The PMLP team finalized her estate planning documents, including drafting a standby-guardianship to protect Ms. A’s son. After Ms. A. signed all the documents, the PMLP team met with both Ms. A and her named fiduciaries to discuss practical considerations for her son after her passing, including decision-making around school, his living situation, and so on. In this way, the PMLP was able to give Ms. A legal stability while her health declined.

### B. Mr. B

Mr. B had fought multiple terminal diagnoses for fifteen years. With no medical path forward, and from his cardiac ICU room late on a Tuesday night in August, Mr. B was ready to address his final affairs. That night, Mr. B and his brother were referred to PMLP. Early on Wednesday morning, the PMLP team met with Mr. B at his bedside, to discuss provisions for his daughter and to execute his will. Mr. B passed away later that day. His brother sent an e-mail that provides insight into the importance and lasting effects of PMLP’s work:

I want to thank you so very much for the wonderful help you gave my brother. After he signed the Will you prepared for him, he felt so much lighter in spirit. And, finally, at peace. The wonderful staff … made [him] feel like he was seen as a person, not just a disease. The kindness, respect, and dignity he was treated with allowed him to blossom in his last days. Having you arrange his affairs allowed him to let go…With a completed Will, every item on his to-do list had been crossed off. Not long after you left, he turned to us and said ‘I’m ready to go.’ He was finally free…Your contribution to his peace of mind was hugely significant. [He] had little to leave his thirteen-year-old daughter, but he was so comforted knowing that everything he did have will go towards her future.

### C. Ms. C

On a Sunday morning, Ms. C, a single mother in her early 40s with terminal cancer, was struggling to breathe. Her 14-year-old son called the ambulance, and he accompanied her to the local hospital emergency department in Northwest Connecticut. Given Ms. C’s rapidly deteriorating condition, the hospital social worker began speaking to Ms. C about the care and custody of her son. This was urgent because if they were not able to make any care arrangements before Ms. C’s death, the Department of Children and Families would take custody of her son. Ms. C mentioned that, prior to her recent discharge from YNHH, she had been working closely with a Smilow social worker and had been referred to PMLP. The social worker then called PMLP, and the team was able to prepare standby guardian documents for Ms. C to sign prior to entering hospice. Ms. C passed a few days later, and because the standby guardian documents were in place, her son was able to move seamlessly into the care of Ms. C’s out-of-state brother.

### D. Common Issues and Observations from the Yale PMLP Experience

Guardianship issues are significant and common in PMLP, and they arise not just in making arrangements for a patient’s own child, but also in determining custodial arrangements for grandchildren and for special-needs family members. Another important issue is educating patients about relevant legal issues from the outset, which can help minimize distress by addressing patients’ worries. For instance, the PMLP team will explain to a patient how advance health care directives and financial powers of attorney work and how these documents can ensure that the person the patient trusts is vested with the power to make decisions and take actions on behalf of the patient.

Moreover, patients themselves do not always know what they need: a patient may think they only need assistance with estate planning, but they actually have additional, sometimes more pressing legal issues, such as a mortgage that must be modified, life insurance beneficiaries to designate, or strong preferences about their funeral arrangements to convey. When lawyers spend enough time with a patient and their family — time to actively listen and assess them holistically from a legal standpoint — the team can offer more comprehensive advice, rather than simply addressing the first issue that a patient raises.

## IV. Special Considerations in PMLP

As the examples above indicate, the situations that arise in PMLP can be very intense, emotional, and sensitive. Issues that come up in many areas of law, like mindful communication, cultural competency, and compassion fatigue, are especially pronounced in the PMLP context. Below, we discuss these considerations in more detail.

### A. Active Listening: Mindful Communication

Active listening is a core skill for all legal professionals and is essential in the PMLP context. “Active listeners assess and accurately allocate resources necessary to the conversation [and] work to create a shared understanding with the speaker by considering both the speaker’s and the listener’s lenses and how they may differ.”^12^ Moreover, active listening requires that the lawyer or student pay attention to both verbal and nonverbal cues and resist the inclination to offer an immediate answer or solution, instead “mov[ing] to a response only after fully exploring and understanding the speaker’s meaning.”^13^ When the patient has a life-limiting illness, and the impact of that illness forms the basis of the professional relationship, establishing trust and understanding the client’s perspective is paramount to successful outcomes.

### B. Cultural Competency

Just as active listening is vital to establishing trust, cultural competency is increasingly important for legal professionals to create effective working relationships. The often invisible lens of a person’s culture “can influence the way one views events; the importance one places on roles, hierarchy or personal relationships; priorities regarding the rights of individuals compared to the group; conflict resolution; emotions and the way emotions are displayed; and one’s willingness to discuss intimate or embarrassing issues among other things.”^14^

Recognition of culture and cultural differences means accepting that “no two people can have exactly the same experiences and thus no two people will interpret or predict in precisely the same ways.” “Culture not only gives us our values and norms of behavior, but also affects how we judge and interact with other people,” and so in PMLP, the team must “gain a client’s trust in a culturally sensitive way.” By emphasizing the importance of cultural competency, lawyers and clients from different backgrounds can create a trusting relationship in which both parties feel comfortable sharing honest and accurate information.

Recognition of culture and cultural differences means accepting that “no two people can have exactly the same experiences and thus no two people will interpret or predict in precisely the same ways.”^15^ “Culture not only gives us our values and norms of behavior, but also affects how we judge and interact with other people,” and so in PMLP, the team must “gain a client’s trust in a culturally sensitive way.”^16^ By emphasizing the importance of cultural competency, lawyers and clients from different backgrounds can create a trusting relationship in which both parties feel comfortable sharing honest and accurate information.

### C. Compassion Fatigue

Lawyers routinely examine difficult, disturbing facts and circumstances — and commonly must do so without any means of working through the effects of such experiences. “Compassion fatigue is associated with the content of the information provided by the client, and the practitioner’s response to the client’s experiences. It is the cumulative physical, emotional, and psychological effect of exposure to traumatic stories.”^17^ The cost of caring in the PMLP becomes very real. The Yale PMLP takes protective efforts to buffer against the long-term effects of compassion fatigue, including routine and substantive collaboration, supportive environments and training, regular encouragement about the importance of self-care, and the setting of reasonable expectations.

### D. When PMLPs Work with Law Students

Law students may not receive much training in active listening before joining a law school’s PMLP, since “[d]espite the importance of active listening for lawyers, legal education has not prioritized the development of this skill.”^18^ PMLP students quickly learn, through firsthand, real-life situations, that the patient is the key storyteller and that they can gain the patient’s trust by understanding the patient’s personal perspective. In clinical work, students will often meet and interact with clients who have very different life experiences, backgrounds, and perspectives from them. Students must learn how to work across differences, meeting clients where they are and listening to and respecting the multiplicity of factors that influence them. “By teaching students cross-cultural lawyering skills and perspectives, we make the invisible more visible and thus help students understand the reactions that they and the legal system may have towards clients and that clients may have towards them.”^19^

### E. Challenges of Client’s Mental Capacity and Who the Client Is

A different kind of challenge in MLP is that of working with patients who have cognitive capacity issues, as many palliative care patients do. Recently, a PMLP law student and legal supervisor spent a great deal of time with a patient doing end-of-life planning, but by the following day, the patient no longer had the capacity to understand or sign any documents. This presents a logistical challenge — how can legal issues be addressed promptly enough when the course of an individual patient’s disease can be unpredictable? — and an emotional one, as despite our best efforts, PMLP may not always be able to ultimately meet the patient’s needs.

Alongside the challenge of unpredictability is an ethical question: who is the client? PMLP often meets with family members alongside patients, who may seek legal advice from us after the patient has passed away. PMLP makes clear to families that the client is the palliative care patient, not their spouse, child, or other family member. Yet when the work provides help and comfort to the family as well as the patient, those lines can become blurry, as “[i]ssues of autonomy and self-determination vs. protection and the best interest of an individual may arise.”^20^ A future area for meaningful legal and ethical consideration is whether there is a workable way to carry over services to any part of the post-death process for family members. Can the scope of PMLP extend beyond the life of a patient to a discrete task, such as a single post-death planning meeting?

## IV. The Challenge of Measuring PMLP

The impact of PMLP on individual clients is significant, yet difficult to quantify. Since programs like PMLP are routinely called upon to demonstrate quantifiable impacts both for scholarship purposes and also for funding, identifying appropriate metrics is an important challenge for the continued development of PMLP programs.

The Yale PMLP saves patients and their families both time and money that would otherwise be spent to hire a private attorney or deal with probate court pro se after the patient dies. The program also saves money for the state when, for example, PMLP completes the necessary steps to place a patient’s child with a family member rather than DCF taking custody after the patient dies. The annual cost to the state of paying a foster family can be more than $10,000 per child;^21^ this number does not consider administrative costs, such as social work involvement and court proceedings. Palliative care clinicians also use metrics such as length of hospital stay, mortality rate, hospital readmission rate, rate of documented treatment preferences, care consistency with documented preferences, pain treatment, and symptom measurements, along with patient and family satisfaction metrics.^22^ However, the most significant impacts are less tangible: how can one put a price on the value of a child staying with their family instead of going into foster care, or the corresponding peace it brings a dying parent to know that their wishes for their child will be followed?

Existential distress is another potentially salient metric. “Existential distress, which overlaps with the concepts of existential suffering, spiritual distress, and demoralization, is distress that arises for patients in the contemplation of their own mortality and is characterized by feelings of helplessness, loneliness, anxiety, and loss of meaning and purpose.”^23^ Existential distress is a significant source of suffering for patients in palliative care who are coming to the end of their lives. In the PMLP context, it can be a source of suffering for the patient’s family members and support network as well, and so the work that the PMLP does can relieve stress and distress on the patient’s entire community of support as well.

The impact of PMLP on a patient’s existential distress is a compelling metric. The challenge is how to measure that impact. The National Hospice and Palliative Care Organization developed the Social Work Assessment Tool, also known as SWAT, “to address the requests of social workers in the hospice and palliative practice arenas to have a quantitative analysis for the effectiveness of social work intervention in patient and family care.”^24^ Palliative care clinicians have recently developed other screening tools as well, such as the Psycho-Existential Symptom Assessment Scale (measuring symptoms such as anxiety, discouragement, hopelessness, and loss of control).^25^ The success of these tools suggests that modeling the tool for use in the PMLP context would be a logical starting point; PMLP could use a rating scale to first measure distress before legal intervention, periodically during the time PMLP is working with the patient, and again after resolving the legal issue.

## Conclusion

Palliative care and MLP share a common mission. By working alongside clinical providers and building trust with the hospital and with patients, PMLP lawyers and law students become valued members of the patient’s care team. PMLP complements palliative care’s holistic approach by helping patients get their legal, custodial, and financial relationships to improve their well-being and offer a higher quality end of life.

## Note

The authors have no conflicts to disclose.
